# Characterization of Rajath Bhasma and Evaluation of Its Toxicity in Zebrafish Embryos and Its Antimicrobial Activity

**DOI:** 10.4014/jmb.1911.11018

**Published:** 2020-03-20

**Authors:** Kalishwaralal Kalimuthu, Ji Min Kim, Chandramohan Subburaman, Woo Young Kwon, Sung Hyun Hwang, Sehan Jeong, Min Geun Jo, Hyung Joo Kim, Ki Soo Park

**Affiliations:** 1 Department of Biological Engineering, College of Engineering, Konkuk University, Seoul 05029, Republic of Korea; 2 Department of Biotechnology, Kalasalingam University, Krishnankoil, India

**Keywords:** Rajath Bhasma, silver, characterization, embryo toxicity, antimicrobial activity

## Abstract

In India, nanotechnology has been used in therapeutic applications for several millennia. One example of a traditional nanomedicine is Rajath Bhasma (also called calcined silver ash), which is used as an antimicrobial and for the treatment of various ailments and conditions such as memory loss, eye diseases, and dehydration. In this study, we aimed to characterize the physical composition and morphology of Rajath Bhasma and its suitability for use as a non-toxic antimicrobial agent. First, Rajath Bhasma was physically characterized via i) Fourier-transform infrared spectroscopy to analyze the surface functional groups, ii) scanning electron microscopy coupled with energydispersive X-ray spectroscopy to observe the morphology and elemental composition, and iii) X-ray diffraction to determine the crystalline phases. Thereafter, functional characterization was performed through toxicity screening using zebrafish embryos and through antimicrobial activity assessment against gram-positive (*Staphylococcus epidermidis*) and gram-negative (*Escherichia coli*) bacteria. Rajath Bhasma was found to harbor alkene, hydroxyl, aldehyde, and amide functional groups originating from biological components on its surface. The main component of Rajath Bhasma is silver, with particle size of 170-210 nm, and existing in the form of spherical aggregates with pure crystalline silver structures. Furthermore, Rajath Bhasma did not exert toxic effects on zebrafish embryos at concentrations below 5 μg/ml and exhibited effective antimicrobial activity against both gram-positive and gram-negative bacteria. The present results indicate that Rajath Bhasma is a potentially effective antimicrobial agent without toxicity when used at concentrations below 5 μg/ml.

## Introduction

Siddha medicine was developed by ancient Tamil sages known as ‘Siddhars’ and has been in use for more than 5,000 years [1]. It involves the use of gold, zinc and silver formulated with honey, ghee, or milk to cure diseases [2, 3]. Prototypical examples include Swarna Bhasma (gold ash), Jasada Bhasma (zinc ash), and Rajath Bhasma (silver ash), which are prepared by processing a fine powder of gold, zinc, and silver with plant extract, followed by repeated incineration at high temperature (~1,000°C). During incineration, the size of the fine powder of gold, zinc, and silver is reduced to that of chemically synthesized nanoparticles (1-100 nm). This fine powder is composed of individual particles of around 50-70 nm in size; however, these particles are prone to forming large aggregates, unlike chemically synthesized nanoparticles [4].

Compared with gold-derived Swarna Bhasma and zinc-derived Jasada Bhasma, the silver-derived medicine Rajath Bhasma has superior and broad-spectrum antimicrobial, antifungal, and antiviral activities [5] and hence numerous applications. Notably, Rajath Bhasma has been used to treat various disorders including anxiety, aging, and infertility [6-8]. The physical characteristics of a silver preparation are believed to markedly influence its therapeutic properties; however, despite its widespread use in India, the exact chemical composition/structure of Rajath Bhasma and its biological activities are largely unknown. Current tools for characterizing these particles at the nanoscale level have furthered the understanding of the parameters directly or indirectly influencing the efficacy of these ancestral medicines. Accordingly, efforts have been made to study these particles at the nanoscale level. For example, Mitra *et al*. [9] have characterized Swarna Bhasma (gold ash) and Jasada Bhasma (zinc ash) and Rohit *et al*. [10] reported the synthesis of Rajath Bhasma through a simple characterization method.

In general, Rajath Bhasma is safe for therapeutic applications [9]; however, in vivo studies are required to accurately predict its toxicity. Zebrafish embryos are a well-established in vivo model for evaluating teratogenicity. Although several studies have investigated the teratogenic effects of silver nanoparticles on zebrafish embryos [11-14], none of them have characterized Rajath Bhasma and assessed its toxicity among embryos at different concentrations to determine the effective concentration for antimicrobial activity.

In this study, we characterized Rajath Bhasma by analyzing its surface functional groups, particle size, elemental composition, and crystalline phase. In addition, we evaluated the toxicity of Rajath Bhasma among zebrafish embryos and its antimicrobial activity in terms of bacterial growth inhibition.

## Materials and Methods

### Characterization of Rajath Bhasma

Rajath Bhasma used in the experiments was purchased from Dabur India Ltd. (India), and analyzed via Fourier-transform infrared (FTIR) spectrometry (Shimadzu 8400; Shimadzu, Japan), scanning electron microscopy (SEM), (EVO 18; Carl Zeiss, Germany), energy-dispersive X-ray (EDX) spectrometry (Quantax 200 with X-Flash; Bruker, USA), dynamic light scattering (DLS) (HORIBA Scientific SZ-100, UK), and X-ray diffraction (XRD),(D8 Advance ECO XRD system with SSD160 1-D detector; Bruker).

### FTIR Analysis

FTIR analysis was performed to determine the surface functional groups of Rajath Bhasma. In brief, a sample was illuminated with infrared light in the wavelength range of 2.5-25 μm, and the transmittance spectrum was obtained to determine the vibrational frequencies corresponding to specific functional groups [15].

### SEM and EDX Analyses

Particle size and shape of Rajath Bhasma were determined via SEM. For SEM analysis, Rajath Bhasma was dispersed in distilled water, sonicated for 2 min, dropped on a carbon tape, and then dried. The elemental composition was assessed using an EDX spectrometer [15].

### DLS Analysis

The average size of Rajath Bhasma was determined via DLS analysis. To determine the size distribution, Rajath Bhasma was dispersed in water for 2 min and the average particle size and standard deviation were determined using the analyzer software.

### XRD Analysis

XRD was used to determine the crystalline phases of Rajath Bhasma. In brief, a thin layer of Rajath Bhasma powder was placed in conventional cavity mounts of the X-ray diffractometer and scanned from 10° to 80° for 2 h. The Cu anode X-ray was operated at 40 kV and 30 mA to generate monochromatic Cu K-α X-rays (k = 1.54056 Å). The average crystallite size of Rajath Bhasma was calculated from the XRD graph using the Debye–Scherrer equation [16]:

$$d=\frac{0.9\lambda }{\mathrm{\beta cos\theta}}$$

where d is the mean crystallite size, λ is the X-ray wavelength, and β is the angular full width at half maximum (FWHM) of the peak at diffraction angle θ.

### Assessment of Embryonic Toxicity

Zebrafish embryos were purchased at a local aquarium shop. All purchases were made in one shop to obtain a relatively uniform group of zebrafish embryos. Zebrafish embryos at the early blastula stage were transferred to a petri plate at 10 embryos/petri plate. These embryos were treated with Rajath Bhasma at different concentrations (5, 10, 15, 20, and 25 μg/ml) in 10 ml distilled water for 48 h at room temperature. At 72 h post fertilization (hpf), malformation rates (%) were calculated as the percentage of dead embryos relative to the total number of embryos to estimate embryonic toxicity [15]. Edema, the most common malformation, along with tail and yolk abnormalities were macroscopically quantified. Each experiment was performed in triplicate [17].

### Assessment of Antimicrobial Activity

The antimicrobial activity of Rajath Bhasma against gram-positive (*Staphylococcus epidermidis*) and gram-negative (*Escherichia coli*) bacteria was determined using the well-diffusion method [18]. In brief, bacteria were diluted to 3.2 × 108 colony forming units/ml and spread on Luria–Bertani agar plates. Thereafter, 6-mm-diameter wells were created in the agar plates by using a sterile borer and 5 μg/ml of Rajath Bhasma was then placed in each well. After incubation at 37°C for 28 h, inhibition zones were measured and the mean of four replicates was calculated.

## Results and Discussion

### Characterization of Rajath Bhasma

**Fig. 1:**
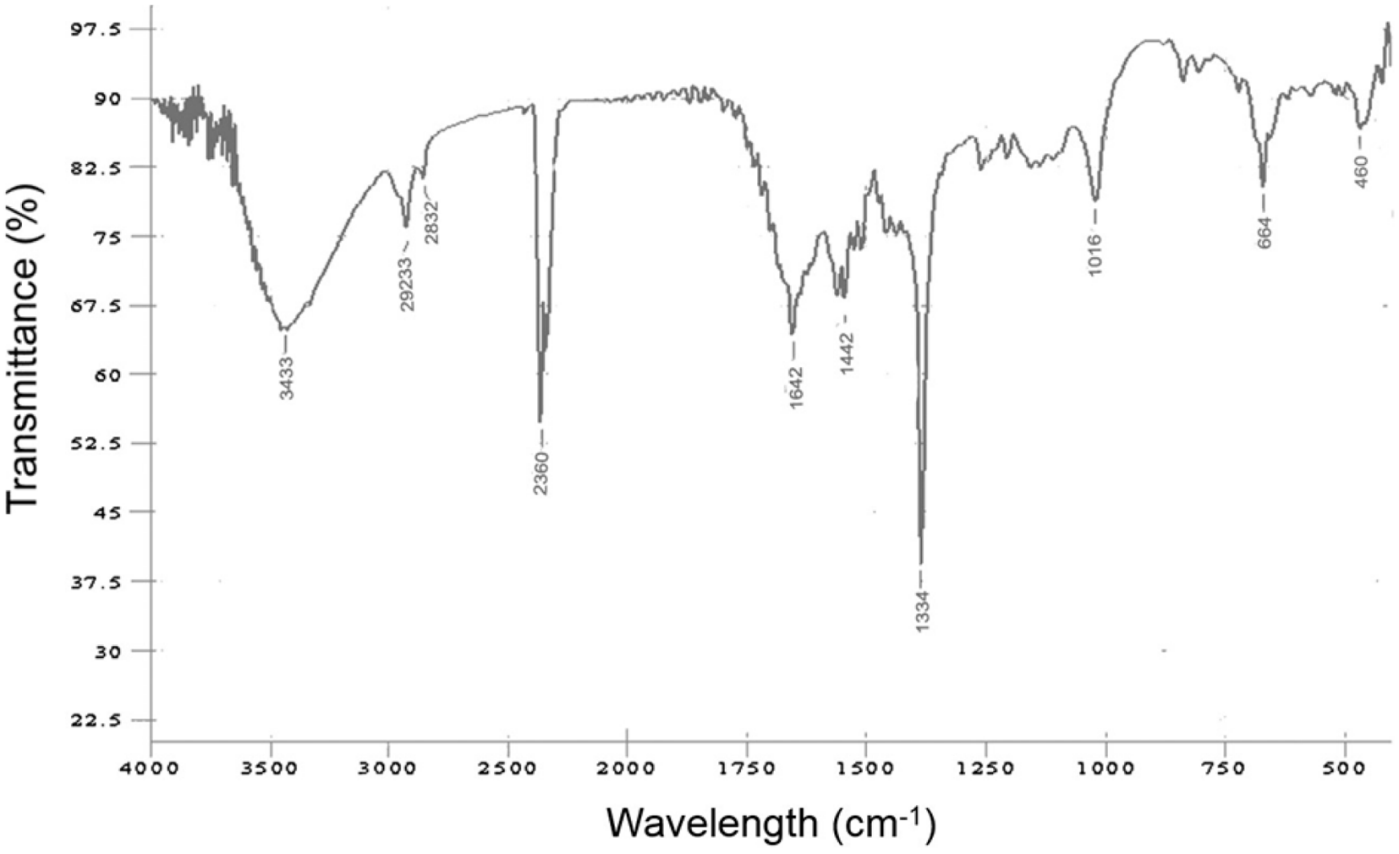
Fourier transform infrared spectroscopy spectrum of Rajath Bhasma.

**FTIR analysis.** Functional groups on the surface of Rajath Bhasma were characterized by studying the vibrational frequencies in the FTIR spectrum (Fig. 1). The broad peak at 3,433 cm^−1^ corresponds to an H-bonded hydroxyl group. The presence of a hydroxyl group is attributed to the preparation method of Rajath Bhasma, in which plant compounds are used to modify the surface of the silver core [19]. The peak at 2,923 cm^−1^ correspond to asymmetric C–H stretching of an aldehyde group and is generally observed in the spectra of biologically synthesized nanoparticles because aldehyde groups are present in the biological component [20]. The sharp peak at 2,360 cm^–1^ corresponds to C=C stretching of the aromatic ring in flavonoids, compounds present in nearly all plants [20]. The peaks at 1,642 cm^–1^ and 1,442 cm^–1^ correspond to C=O stretching and CH_2_ bending vibrations of aliphatic compounds, respectively [21, 22]. The major stretching peak at 1,334 cm^–1^ is assigned to amide groups, which are abundant in plant extracts [23]. Together, these results indicate that the surface of the silver core is adorned with organic compounds including plant or herbal extracts.

**Fig. 2:**
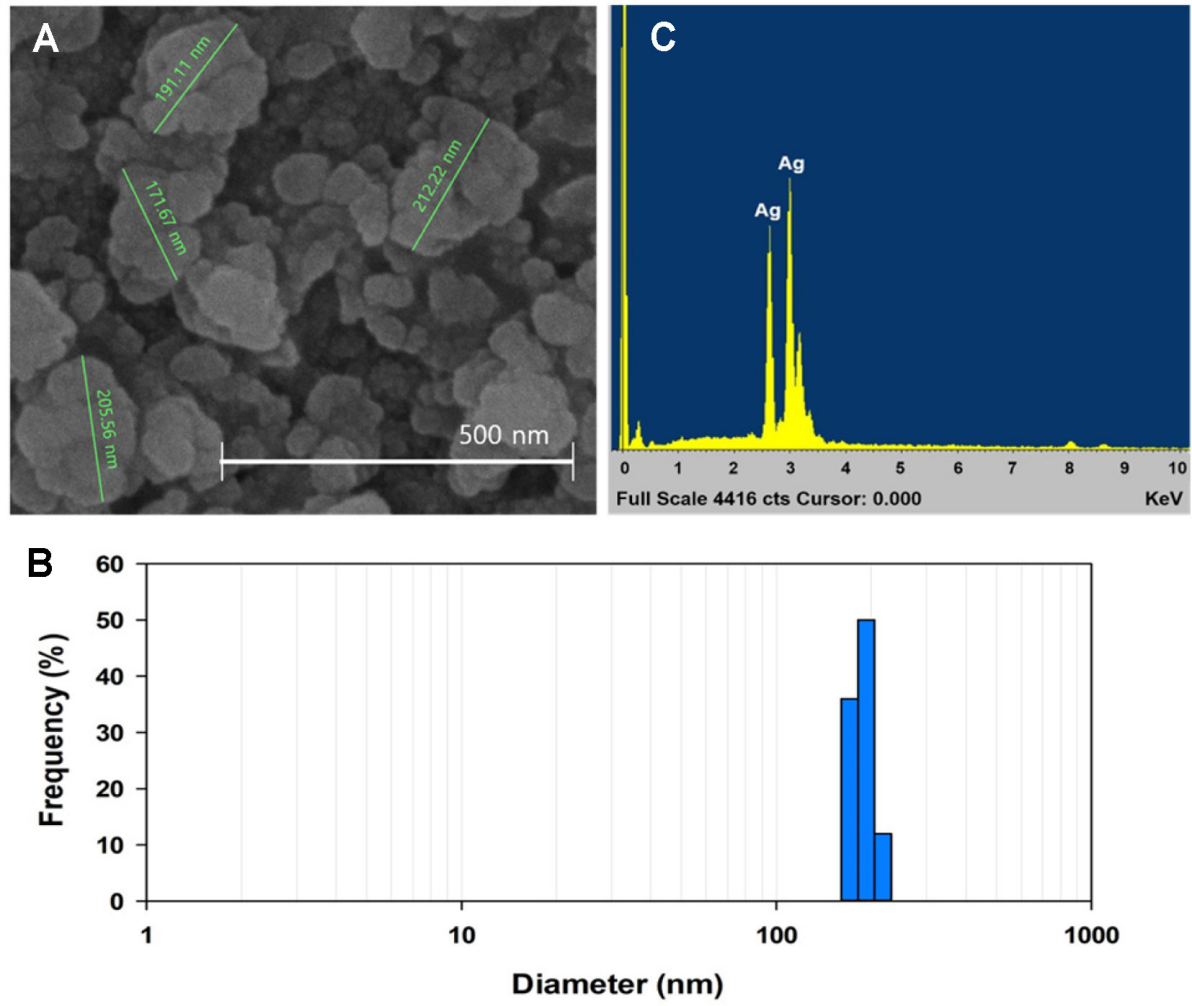
Scanning electron microscope image (A), dynamic light scattering graph (B) and energy-dispersive X-ray graph (C) of Rajath Bhasma.

**SEM and EDX analyses.** The particle size of Rajath Bhasma was analyzed via SEM. Fig. 2A shows that the nanoparticles formed spherical aggregates, and the size of individual aggregates ranged from 170 to 210 nm, consistent with the results of DLS analysis (Fig. 2B). In addition, EDX analysis confirmed that silver is the major component (approximately 79%) of Rajath Bhasma (Fig. 2C).

**Fig. 3:**
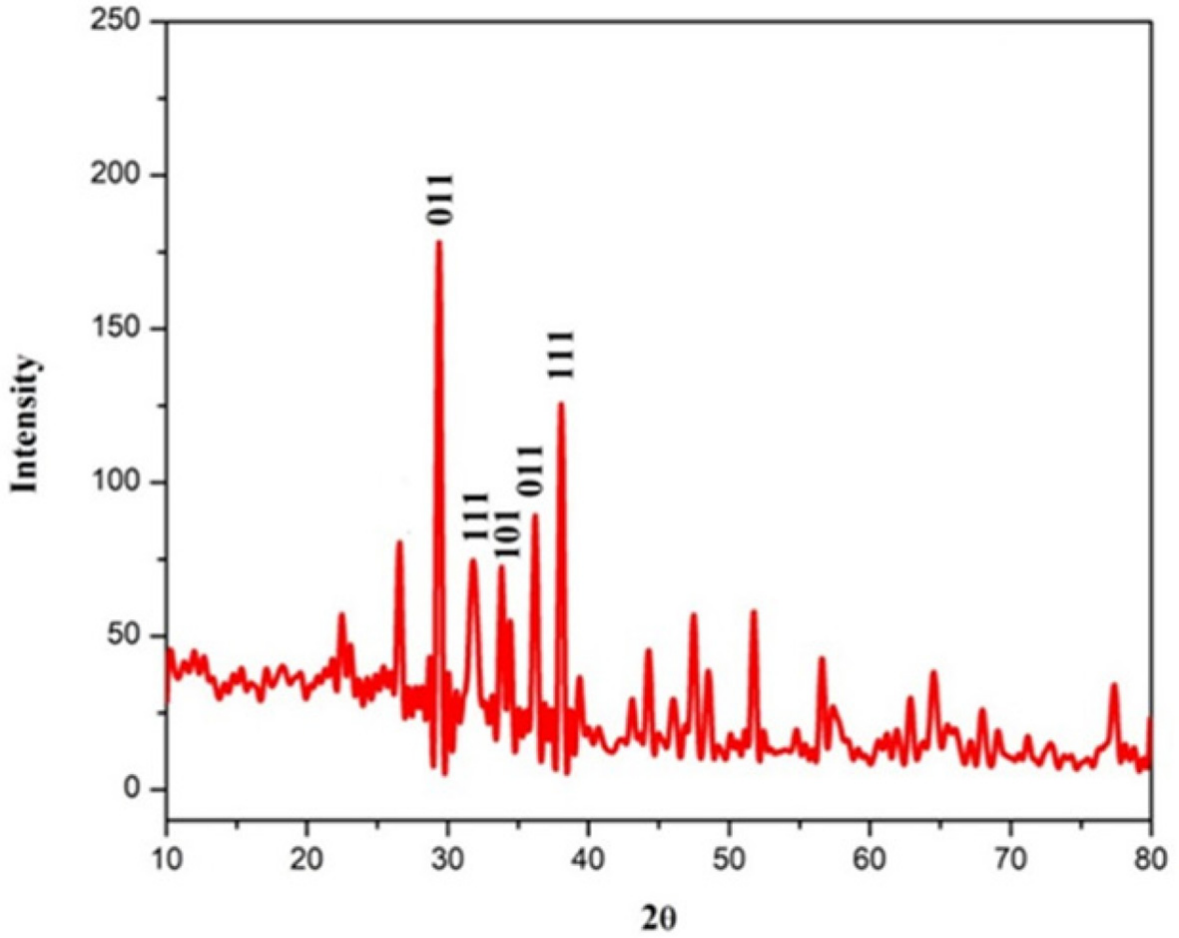
X-ray diffraction spectrum of Rajath Bhasma.

**XRD analysis.** XRD analysis was performed to identify the crystalline phases in Rajath Bhasma. The XRD pattern showing sharp diffraction peaks at 29.79°, 32.28°, 33.64°, and 38.116°, which correspond to crystalline planes of (011), (111), (101), and (111), respectively, indicated pure crystalline silver structures (Fig. 3). The XRD results were concurrent with the Joint Committee on Powder Diffraction Standards (JCPDS) data for silver oxalate (Ag_2_C_2_O_4_; JCPDS 22-1335). The peaks at 32.28 (111), 33.64 (101), and 38.116 (111) also correspond to AgO (JCPDS 84-1547), Ag_2_CO_3_ (JCPDS 71-2184), and Ag (JCPDS 65-2871), respectively. The average crystallite size of Rajath Bhasma was calculated using the Debye–Scherrer equation. Table 1 shows that the average crystallite size was approximately 55 nm [24-26].

### Toxic Effects of Rajath Bhasma on Zebrafish Embryos

**Fig. 4:**
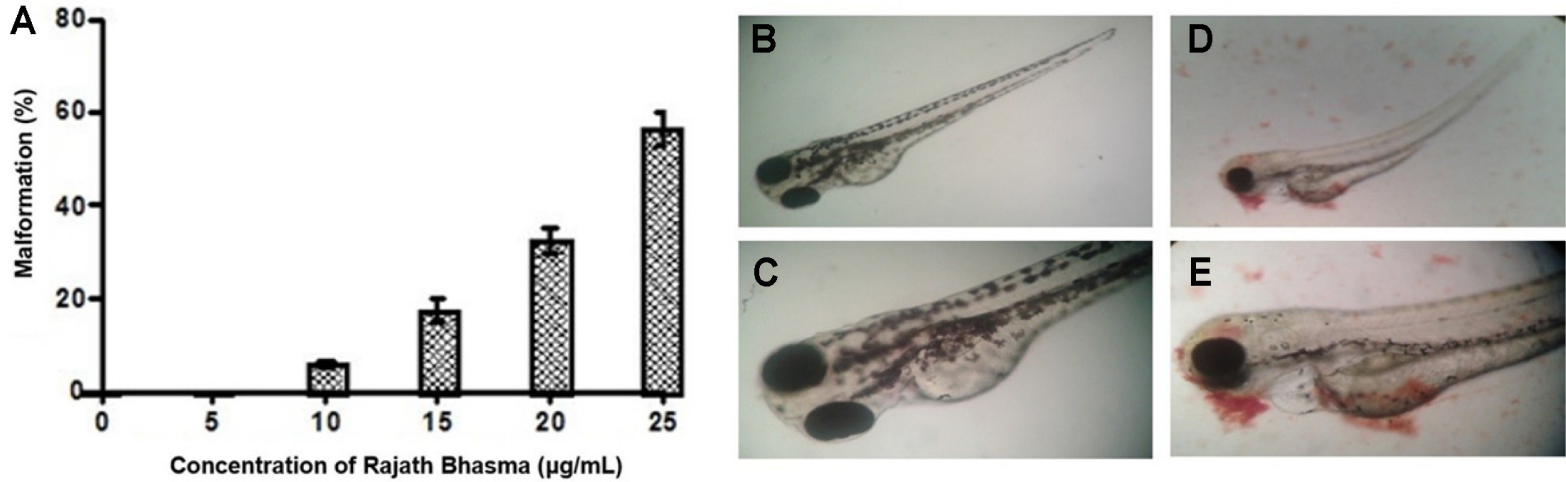
Zebrafish embryo toxicity of Rajath Bhasma. (**A**) Malformation rates of zebrafish embryos at different concentrations of Rajath Bhasma. The photographs show zebrafish embryos in the absence of Rajath Bhasma (**B**, **C**) and embryos exposed to 25 μg/ml Rajath Bhasma (**D**, **E**).

Zebrafish embryos at an early blastula stage were exposed to Rajath Bhasma for 48 h and embryonic malformation rates were determined at 72 hpf. As shown in Fig. 4A, 5 μg/ml of Rajath Bhasma did not substantially inhibit the growth of zebrafish embryos [16]. However, the malformation rate was approximately 60% at 25 μg/ml (Fig. 4). These results indicate the importance of determining optimal therapeutic concentrations to inhibit bacterial growth without inducing developmental toxicity in zebrafish.

### Antimicrobial Activity of Rajath Bhasma against *S. epidermidis* and *E. coli*

**Fig. 5:**
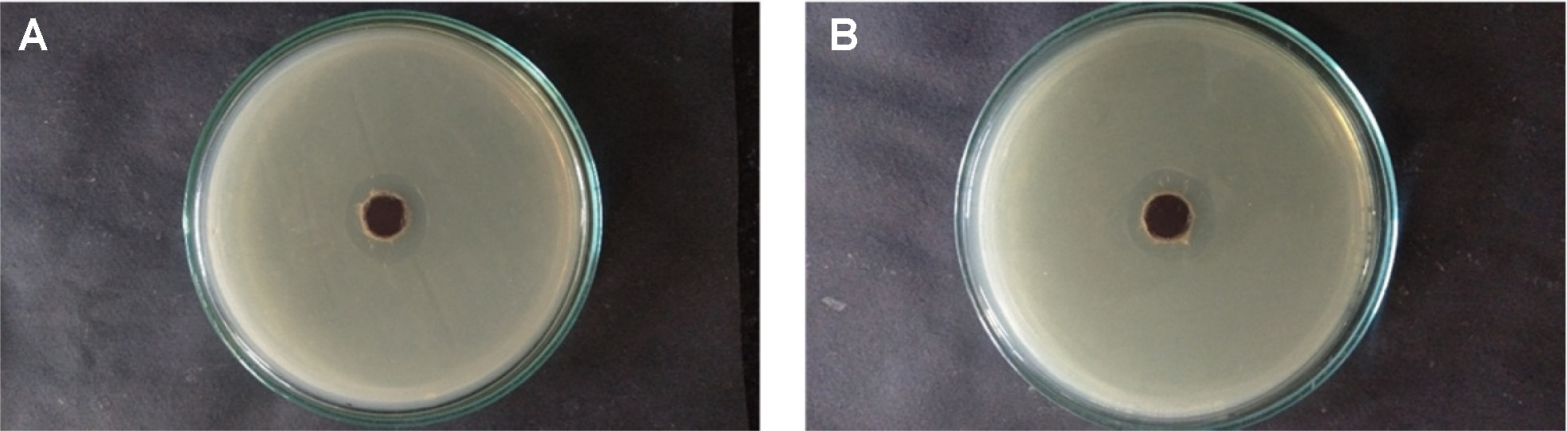
Representative results of the assessment of antimicrobial activity of Rajath bhasma (5 μg/ml) against *Staphylococcus epidermidis* (A) and *Escherichia coli* (B).

Silver nanoparticles are one of the most effective antimicrobial agents; thus, the antimicrobial activity of Rajath Bhasma was investigated by assessing its antibacterial effect. Inhibition zones were clearly observed for both gram-positive and gram-negative bacteria. The inhibition zone for *S. epidermidis* treated with 5 μg/ml Rajath Bhasma (*i.e.*, the concentration at which no embryo toxicity was observed; Fig. 4) was 4 ± 0.5 mm and that for *E. coli* was 5 ± 0.5 mm, confirming its effective antimicrobial activity (Fig. 5). Notably, the growth of gram-positive bacteria, which have a thick peptidoglycan layer, was inhibited as effectively as that of gram-negative bacteria. This was attributed to the presence of proton-donating hydroxyl and amide groups on the surface of Rajath Bhasma, as evident from FTIR (Fig. 1) [10].

In conclusion, we characterized Rajath Bhasma using FTIR, SEM, DLS, EDX, and XRD to determine the factors affecting its biological activities and functions. We identified various types of surface functional groups derived from organic components and we confirmed that silver nanoparticles, with a size range of 170–210 nm, are the major component in Rajath Bhasma. Our results show that Rajath Bhasma is a potentially effective antimicrobial agent without toxicity when used at a low concentration (<5 μg/ml). To our knowledge, this is the first study to evaluate the chemical properties, antimicrobial activity, and zebrafish embryonic toxicity of Rajath Bhasma. Our findings will not only expand the application range of Rajath Bhasma but also guide future studies aimed at developing novel nanomaterials.

## Figures and Tables

**Table 1:** Crystallite sizes of Rajath Bhasma determined via XRD analysis.

2θ	FWHM (dθ)	Crystallite size (nm)
36.2106	0.2085	63.2
38.0529	0.1601	81.8
45.9921	0.8829	14.46
47.4855	0.2712	46.7
51.7401	0.1799	69.3

XRD, X-ray diffraction; FWHM, angular full width at half maximum.

## References

[1] Kalimuthu Kalishwaralal, Venkataraman Deepak, SureshBabu Ram Kumar Pandian, Muniasamy Kottaisamy, Selvaraj BarathmaniKanth, Bose Kartikeyan (2010). Biosynthesis of silver and gold nanoparticles using Brevibacterium casei. Colloids Surf. B Biointerfaces.

[2] Royan CU (1957). Siddha hospital pharmacopeia.

[3] Fritts M, Crawford CC, Quibell D, Gupta A, Jonas WB, Coulter I (2008). Traditional Indian medicine and homeopathy for HIV/AIDS: A review of the literature. AIDS Res. Ther..

[4] Daniel Beaudet, Simona Badilescu, Kiran Kuruvinashetti, Ahmad Sohrabi Kashani, Dilan Jaunky, Sylvie Ouellette (2017). Comparative study on cellular entry of incinerated ancient gold particles (Swarna Bhasma) and chemically synthesized gold particles. Sci. Rep..

[5] Wijnhoven SWP, Peijnenburg WJGM, Herberts CA, Hagens WI, Oomen AG, Heugens EHW (2009). Nano-silver - a review of available data and knowledge gaps in human and environmental risk assessment. Nanotoxicology.

[6] Nagarajan S, Krishnaswamy S, Pemiah B, Rajan KS, Krishnan U, Sethuraman S (2014). Scientific insights in the preparation and characterisation of a lead-based *Naga Bhasma*. Indian J. Pharm. Sci..

[7] Bagul MS, Kanaki NS, Rajani M (2005). Evaluation of free radical scavenging properties of two classical polyherbal formulations. Indian J. Exp. Biol..

[8] Donga SB, Parikh P, Induben U (2012). PA01.45. An ayurvedic management of vandhyatva w.s.r. to cervical factor. Anc. Sci. Life.

[9] Mitra A, Chakraborty S, Auddy B, Tripathi P, Sen S, Saha AV (2002). Evaluation of chemical constituents and free-radical scavenging activity of Swarnabhasma (gold ash), an ayurvedic drug. J. Ethnopharmacol..

[10] Hamouda RA, Hussein MH, Abo-elmagd RA, Bawazir SS (2019). Synthesis and biological characterization of silver nanoparticles derived from the Cyanobacterium Oscillatoria limnetica. Sci. Rep..

[11] AshaRani PV, Mun GLK, Hande MP, Valiyaveettil S (2009). Cytotoxicity and genotoxicity of silver nanoparticles in human cells. ACS Nano.

[12] Bilberg K, Hovgaard MB, Besenbacher F, Baatrup E (2012). In vivo toxicity of silver nanoparticles and silver ions in zebrafish (Danio rerio). J. Toxicol..

[13] Yoo MH, Rah YC, Choi J, Park S, Park H-C, Oh KH (2016). Embryotoxicity and hair cell toxicity of silver nanoparticles in zebrafish embryos. Int. J. Pediatr. Otorhinolaryngol..

[14] Liu X, Dumitrescu E, Kumar A, Austin D, Goia D, Wallace K (2019). Differential lethal and sublethal effects in embryonic zebrafish exposed to different sizes of silver nanoparticles. Environ. Pollut..

[15] Kalishwaralal K, Jeyabharathi S, Sundar K, Muthukumaran A (2016). A novel one-pot green synthesis of selenium nanoparticles and evaluation of its toxicity in zebrafish embryos. Artif. Cells Nanomed. Biotechnol..

[16] Kalishwaralal K, Deepak V, Ramkumarpandian S, Nellaiah H, Sangiliyandi G (2008). Extracellular biosynthesis of silver nanoparticles by the culture supernatant of *Bacillus licheniformis*. Mater. Lett..

[17] María E Díaz-Casado, Iryna Rusanova, Paula Aranda, Marisol Fernández-Ortiz, Ramy K A Sayed, Beatriz I Fernández-Gil (2018). In vivo determination of mitochondrial respiration in 1-methyl-4-phenyl-1,2,3,6-tetrahydropyridine-treated zebrafish reveals the efficacy of melatonin in restoring mitochondrial normalcy. Zebrafish.

[18] Kalimuthu Kalishwaralal, Selvaraj BarathManiKanth, Sureshbabu Ram Kumar Pandian, Venkataraman Deepak, Sangiliyandi Gurunathan (2010). Silver nanoparticles impede the biofilm formation by *Pseudomonas aeruginosa* and *Staphylococcus epidermidis*. Colloids Surf. B Biointerfaces.

[19] Chaturvedi R, Jha C (2011). Standard manufacturing procedure of Rajata Bhasma. Ayu.

[20] Peng Wu, Wei Li, Qiong Wu, Yushan Liu, Shouxin Liu (2017). Hydrothermal synthesis of nitrogen-doped carbon quantum dots from microcrystalline cellulose for the detection of Fe^3+^ ions in an acidic environment. RSC Adv..

[21] Mahadevan S, Vijayakumar S, Arulmozhi, P (2017). Green synthesis of silver nano particles from Atalantia monophylla (L) Correa leaf extract, their antimicrobial activity and sensing capability of H_2_O_2_. Microb. athog..

[22] Nakason K, Panyapinyopol B, Kanokkantapong V, Virya-empikul N, Kraithong W, Pavasant P (2018). Hydrothermal carbonization of unwanted biomass materials: Effect of process temperature and retention time on hydrochar and liquid fraction. J. Energy Inst..

[23] Baia TC, Gama RA, Silva De Lima LA, Lima KMG (2016). FTIR microspectroscopy coupled with variable selection methods for the identification of flunitrazepam in necrophagous flies. Anal. Methods.

[24] Bharani M, Thirunethiran Karpagam, Varalakshmi B, Indria S (2012). Synthesis and characterization of silver nano particles from wrightia tinctoria. Int. J. Appl. Biol. Pharm. Tech..

[25] Wojnarowicz J, Opalinska A, Chudoba T, Grerlotka S, Mukhovskyi, Pierzylowska (2016). Effect of water content in ethylene glycol solvent on the size of ZnO nanoparticles prepared using microwave solvothermal synthesis. J. Nanomater..

[26] Hiremath R, Jha CB, Narang KK (2010). Vanga Bhasma and its XRD analysis. Ancient. Sci. Life..

